# Is cognitive behavioral therapy a better choice for women with postnatal depression? A systematic review and meta-analysis

**DOI:** 10.1371/journal.pone.0205243

**Published:** 2018-10-15

**Authors:** Lili Huang, Yunzhi Zhao, Chunfang Qiang, Bozhen Fan

**Affiliations:** Department of Gynaecology and Obstetrics, Putuo Hospital, Shanghai University of Traditional Chinese Medicine, Shanghai, China; Chiba University Graduate School of Medicine, JAPAN

## Abstract

The present study evaluated the combined effectiveness of cognitive behavioral therapy (CBT) for postnatal depression. A systematic search was conducted across databases including PubMed, Embase, and the Cochrane library to identify the randomized controlled trials (RCTs) that assessing CBT versus control for postnatal depression until March 2017. Data was extracted by two reviewers, independently. The Review Manager 5.3 and Stata 11.0 were used to calculate the synthesized effect of CBT on depression, and anxiety. A total of 20 RCTs involving 3623 participants were included. The results of meta-analysis showed that CBT was associated with a better Edinburgh Postnatal Depression Scale (EPDS) than control in short-term (mean difference = -2.86, 95% CI: -4.41–-1.31; *P*<0.05) and long-term (mean difference = -1.68, 95% CI: -1.81–1.56; *P*<0.05). CBT also improved short-term (mean difference = -6.30, 95% CI: -11.32–-1.28; *P*<0.05) and long-term (mean difference = -4.31, 95% CI: -6.92–-1.70; *P*<0.05) Beck Depression Inventory (BDI). Subgroup analysis based on intervention types showed that in-home and telephone-based therapy exhibited significant reductions in EPDS scores (*P*<0.05 for all). CBT significantly improved the short-term [odds ratio (OR) = 6.57, 95% CI: 1.84–23.48; *P*<0.05] and long-term (OR = 2.00, 95% CI: 1.61–2.48; *P*<0.05) depressive symptomatology as compared to control. CBT also reduced the score of Depression Anxiety Stress Scales (DASS), though without significance. In conclusion, CBT effectively improved the symptoms and progression of postnatal depression.

## Introduction

Pregnancy is usually accompanied with various psychological conditions such as depression, even after childbirth [1]. As reported by the world health organization (WHO), depression is a leading cause of disability worldwide [2]. Depression is rendered to increase the disease burden in women with respect to all health conditions [3]. Depression negatively affects physical and social functions and is a major risk factor for suicide [4]. Anxiety and depression are the most common symptoms in the complex psychological changes during pregnancy [5–7]. Physical, pathological, and social changes, that leading to anxiety and depression will continue from the prenatal period to puerperium [8, 9]. Thus, the most common female mental disorder, that is postpartum depression, refers to depression occurring during puerperium. Postpartum depression usually occurs 2 weeks after childbirth, and the symptoms are typical at 4–6 weeks [8, 9]. Postnatal depression (PND) is often referred to a condition occurring after childbirth [10]. In most studies, the population of PND includes women with continuous depression from pregnancy to childbirth, as well as women who never experienced depression until childbirth [2, 10]. Previous studies also reported that the prevalence of PND ranged from 13% to 19.2% in new mothers during the first few months after childbirth [11, 12]. The common symptoms of PND include emotional lability, guilt, dysphoria, confusion, and even suicidal tendency [4, 13]. If untreated, severe clinical symptoms will appear and ultimately increase the risk of suicide [4, 13]. Meanwhile, some studies suggested that early diagnosis and intervention of PND are advantageous in relieving the negative impacts on women [3, 14].

A variety of PND treatment options, including a combination of therapies encompassing medication and psychotherapy exist [5]. Antidepressants such as tricyclic antidepressants, monoamine oxidase inhibitors, and selective 5-hydroxytryptamine (5-HT) reuptake inhibitors can effectively improve the symptoms of PND. However, the medication therapy may affect the baby via milk metabolism [15, 16]. Thus, clinical attention has been focused on psychological counseling and interventions. A number of published trials have compared the efficacy of different therapies on PND [3, 15, 17]. The psychological therapy can stimulate the inherent maternal motivation, improve the ability to cope with PND without carrying adverse drug reactions to breastfeed infants [18, 19]. Investigators speculated that abnormal emotions and behavior of PND were related to distorted cognition, thereby recommending psychological treatment as the preferred approach for PND [20, 21]. Besides, PND patients prefer psychological therapies, such as cognitive behavioral therapy (CBT), psychodynamic therapy, interpersonal therapy (IPT), and counseling [2, 19]. Some reviews have shown that these means are effective options for improving PND [2, 22]. CBT is one of the non-pharmacological therapies that has been applied in clinical practive in recent years [19, 23]. Several studies have reported that CBT exerts significant therapeutic effects on PND [2, 8, 11, 23, 24]. Whether CBT and which type of CBT is superior in reducing risk and relieving symptoms of PND is still required to be elucidated.

In this study, we aimed to explore whether CBT was more efficient in improving the depressive condition of women following childbirth as compared to treatment as usual by meta-analysis.

## Materials and methods

This study was performed based on the Preferred Reporting Items for Systematic Reviews and Meta-Analyses (PRISMA) guidelines (S1 Table).

### Search strategy

Databases including PubMed, Embase, and the Cochrane Library were systematically searched for identifying randomized controlled trials(RCT) with respect to CBT in women with PND until March 2017. Additional relevant studies were also screened from references of the included studies, conference or meeting abstracts, and registered websites of clinical trials. The keywords and terms such as “puerperal depression”, “postnatal depression”, “postpartum depression”, “puerperium depression”, “cognitive-behavioral”, and “cognitive behavioral therapy” were used in various combinations during the search. The number of selected studies in different databases were recorded in Excel table. No regional and racial restrictions were applied during the search. The primary language of eligible studies was English; however, articles in Chinese or other languages were also considered for inclusion as necessary. The manually searching of target journals was performed to obtain potential eligible studies and the corresponding authors of included studies were contacted if there were missing key data.

### Inclusion and exclusion criteria

Inclusion criteria: Patients: The cohort of patients included women, who gave birth to an infant in the past year, with formal diagnosis of PND or with the risk of PND. Comparison: Each trial was comprised of two groups, one received CBT, and the other received usual care, home visiting, waitlist control, or other conventional treatment. Outcomes: The mean depression score and its associated standard deviation before and after intervention in the control and experimental groups were extracted and recorded[common depression score scale includes Edinburgh Postnatal Depression Scale (EPDS) and Beck Depression Inventory (BDI-II)] [9, 25]. The improvement rates of PND after treatments were also extracted. Study design: the forms of CBT included internet-based therapy, telephone-based therapy, in-home therapy, and mindfulness-based cognitive behavioral therapy. Therefore, if the interventions of the included studies belonged to one of the therapies mentioned above, the RCTs would be considered for inclusion.

Exclusion criteria: Observational studies, animal studies, reviews, and other types of non-RCTs were excluded. Articles without sufficient information or data were also discarded after contacting key authors.

### Data extraction

The baseline characteristics of patients and target data of outcomes were extracted from eligible studies by two reviewers, independently. These information were about study authors, published year, the number of cases, average age, sex ratio, diagnostic criteria of PND, and baseline history of depression. Primary data were interventions, symptoms of anxiety and depression, improvements rates, different types of depression score scale, diagnostic accuracy statistics, relapse rate of PND. Secondary outcome was the improvement in quality of life. When there was discrepancy about the extracted data, it would be resolved by discussing with the third reviewer.

### Quality assessment of included studies

RCTs were included only if they fulfilled the inclusion criteria and the following quality assessment. The quality of included studies was evaluated according to the criteria provided by the Cochrane Handbook for Systematic Reviews of Interventions [26]. The quality elements such as selection bias, blinding bias, incomplete outcome data, selective reporting bias, and other bias were assessed. A third reviewer resolved the disagreement in case of controversy. The final quality of each study was defined as high-risk, unclear risk or low-risk of bias, according to the handbook mentioned above.

### Statistical analysis

This meta-analysis was conducted using the RevMan 5.3 software provided by the Collaboration Network [26] and the Stata 11.0 software (StataCorp LLC). Data were represented as standardized mean difference (SMD) and its 95% confidence interval (95% CI) [2]. The chi-squared test (presented as I^2^) was used to analyze the heterogeneity of the included studies (*P*<0.1 was set as the test level), and the degree of heterogeneity was judged based on the value of I^2^. If I^2^ was >50%, it was considered a high-risk of heterogeneity. Then, the subgroup analysis was introduced to evaluate the clinical homogeneity. If a high-risk of heterogeneity as absent in the analysis, the fixed effect model was used, otherwise, the random-effects model was applied. The sensitivity analysis was conducted when a high-risk of bias was indicated. The odds ratio (OR) with a 95% confidence interval (95% CI) was used to present the dichotomous data. The continuous variables were analyzed in term of mean ± standard deviation. For continuous data, the weighted mean difference (WMD) was used if the measurement unit of continuous variables was identical, or, the standardized mean difference (SMD) was used. Funnel plots and the Egger’s and Begg’s tests were used to assess the publication bias. A *P*<0.05 was considered statistically significant for combined effectiveness assessment.

## Results

### Literature research

**Fig 1:**
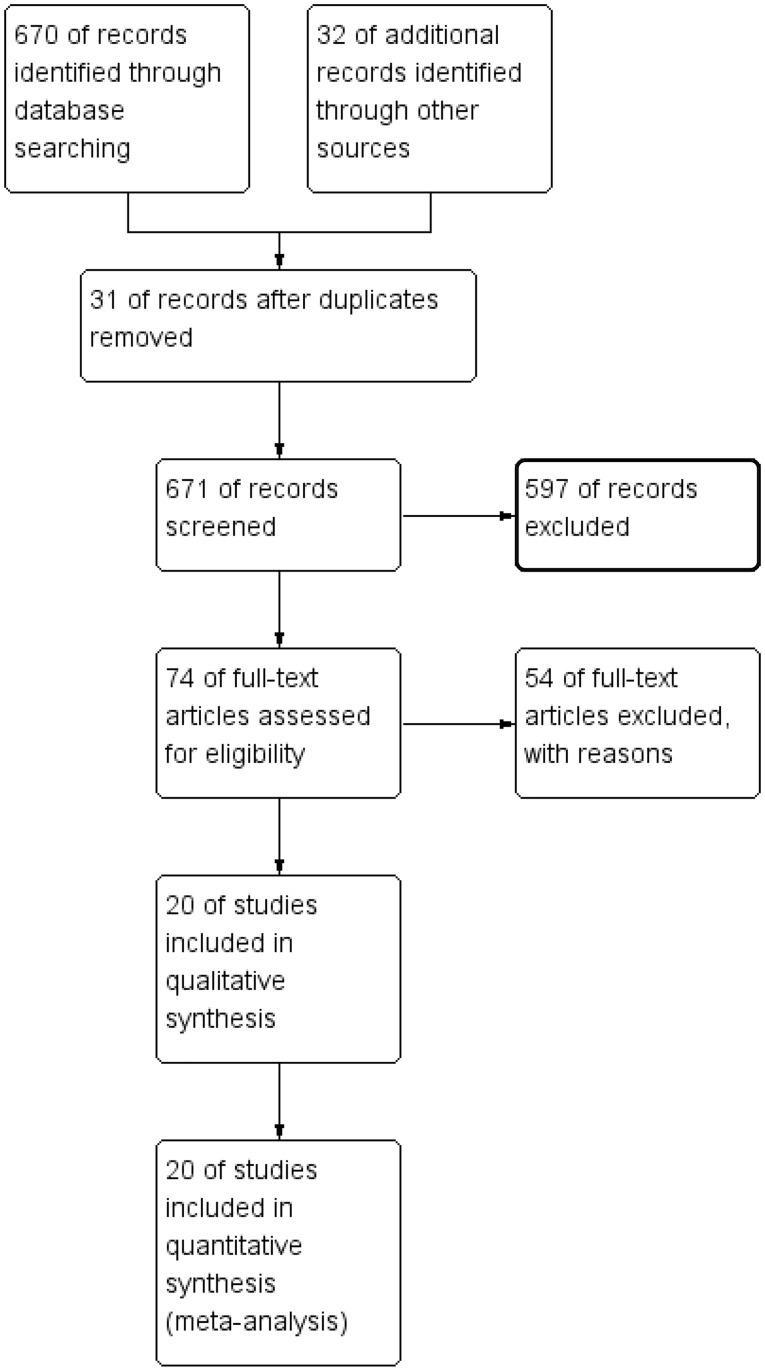
Schematic of the study selection.

The search results of the RCTs by the two evaluators were highly consistent. According to the search strategy and data collection methods, a total of 702 articles published from 1993–2017 were retrieved. After screening the titles and abstracts, 628 articles were abandoned due to the irrelevant topic or research purposes, animal study, drug treatment, or improper evaluation of efficiencies and outcomes. After further reading the full text, 54 studies were excluded because of non-RCTs, reviews, and reduplication. Finally, 20 studies [15, 20, 21, 27–43] (Fig 1) were included. These studies involved 3623 participants, comprising of 1923 cases in the psychological intervention group and 1700 in the control group. These studies were performed worldwide and published in English.

### General information and characteristics of included studies

#### Patients

Among these 3623 participants, 1923 cases were in the intervention group, and the remaining 1700 cases were in the control group. The age of this population ranged from 18 to 37 years. Most of the participants were primiparous and married. The definition and criteria of PND varied among the included studies. EPDS, BDI, Beck Anxiety Inventory (BAI), and Hamilton Rating Scale were used in various studies for defining PND. The baseline characteristics of the eligible studies are listed in Table 1.

**Table 1:** Baseline characteristics of included studies.

Author	Year	Region	Types of CBT	Int	Con	total number	N in Int	N in Con	Age in Int	Age in Con	Outcomes	measure methods
Ngai FW	2017	China	telephone	CBT	Treatment as usual	397	197	200	31.1 (3.8)	30.4 (4.4)	Health related QOL	SF-12
Pugh NE	2016	Canada	internet	CBT	Treatment as usual	47	24	23	>18	>18	depression, recovery	EPDS, PSI, DASS
Leung SS	2016	China	group	CBT	Treatment as usual	164	82	82	31.56(3.78)	30.9(5.7)	depression, anxiety	EPDS, HADS
Dimidjian S	2016	USA	mindfulness	CBT	Treatment as usual	86	43	43	30.98 (4.08)	28.72 (5.50)	Depressive Symptom Severity	EPDS
Milgrom J	2016	Australia	internet	CBT	Treatment as usual	43	21	22	31.7(4.6)	31.5(4.3)	depression, anxiety	SCID, BDI, DASS
Fathi-Ashtiani A	2015	Iran	hospital	CBT	Treatment as usual	135	64	71	25(3.58)	26(3.82)	depresssion, anxiety	EPDS, BDI, BAI
Ngai FW	2015	China	telephone	CBT	Treatment as usual	397	197	200	31.1(3.8)	30.4(4.4)	depression, recovery	EPDS
Milgrom J	2015	Australia	group	CBT	Treatment as usual	45	30	15	30.1 (5.5)	31.6 (3.2)	depression, anxiety	BDI,BAI,PSI
Hou Y	2014	China	hospital	CBT	Treatment as usual	249	128	121	28(3)	28(4)	depression and sleep quality	EPDS, PSQI
O’Mahen HA	2013	UK	internet	CBT	Treatment as usual	910	462	448	32.3 (4.7)	32.2 (5.7)	depresssion, recovery	EPDS
Ammerman RT	2013	USA	in-home	CBT	standard home visiting	93	47	46	22.4 (5.2)	21.5 (3.9)	psychological distress, social support, and social network	Brief Symptom Inventory
Milgrom	2011	Australia	group	CBT	General practitioner management	68	22/23	23	31.5 (4.7)	31.5 (4.7)	depression, recovery	BDI
Morrell	2009	UK	in-home	CBT	Treatment as usual	418	271	147	30.9 (5.4)	30.9 (5.4)	depression, recovery	EPDS, STAI, SF-12, PSI
Austin MP	2007	Australia	group	CBT	Treatment as usual	277	191	86	31.4	31.4	depression, anxiety	EPDS, STAI
Milgrom	2005	Australia	group	CBT	Treatment as usual	192	46	33	29.7 (5.4)	29.7 (5.4)	depression	BDI, BAI, social support
Misri S	2004	USA	group	CBT	Treatment as usual	35	19	16	29.52 (5.85)	30.81 (3.31)	depression, anxiety	HAM-A, HAM-D, EPDS
Cooper	2003	UK	in-home	CBT	Treatment as usual	193	43	52	27.7 (5.4)	27.7 (5.4)	depression, anxiety	EPDS
Honey	2002	UK	hospital	CBT	Treatment as usual	45	23	22	27.9 (5.5)	27.9 (5.5)	Depressive Symptom	EPDS
Chabrol H	2002	France	in-home	CBT	Treatment as usual	48	18	30	30.5 (4.3)	30 (5)	depression, recovery	BDI,EPDS
Prendergast	2001	USA	group	CBT	Ideal standardized care	37	17	20	32.2	32.2	depression	EPDS

Abbreviations: CBT, cognitive behavioral therapy; Int, intervention; Con, control; N, number; QOL, quality of life; EPDS, Edinburgh Postnatal Depression Scale; PSI, Parenting Stress Index; DASS, Depression Anxiety Stress Scales; BDI, Beck Depression Inventory; HADS, Hospital Anxiety and Depression Scale; BAI, Beck Anxiety Inventory; PSQI, Pittsburgh Sleep Quality Index; HAM-A, 14-item Hamilton Rating Scale for Anxiety; HAM-D, 21-item Hamilton Rating Scale for Depression; STAI, State-Trait Anxiety Inventory.

### Interventions

All studies had intervention and control arms. Among these studies, internet-based therapy, telephone-based therapy, in-home therapy, and mindfulness therapy were used as the intervention for PND patients. These therapies were carried out by psychologists, or trained nurses, or researchers. The duration of treatment ranged from 1 to 12 sessions. On the other hand, traditional treatments such as medication, counseling service, collaborative care, and other forms of therapies were used in the control groups.

### Quality assessment of included studies

**Fig 2:**
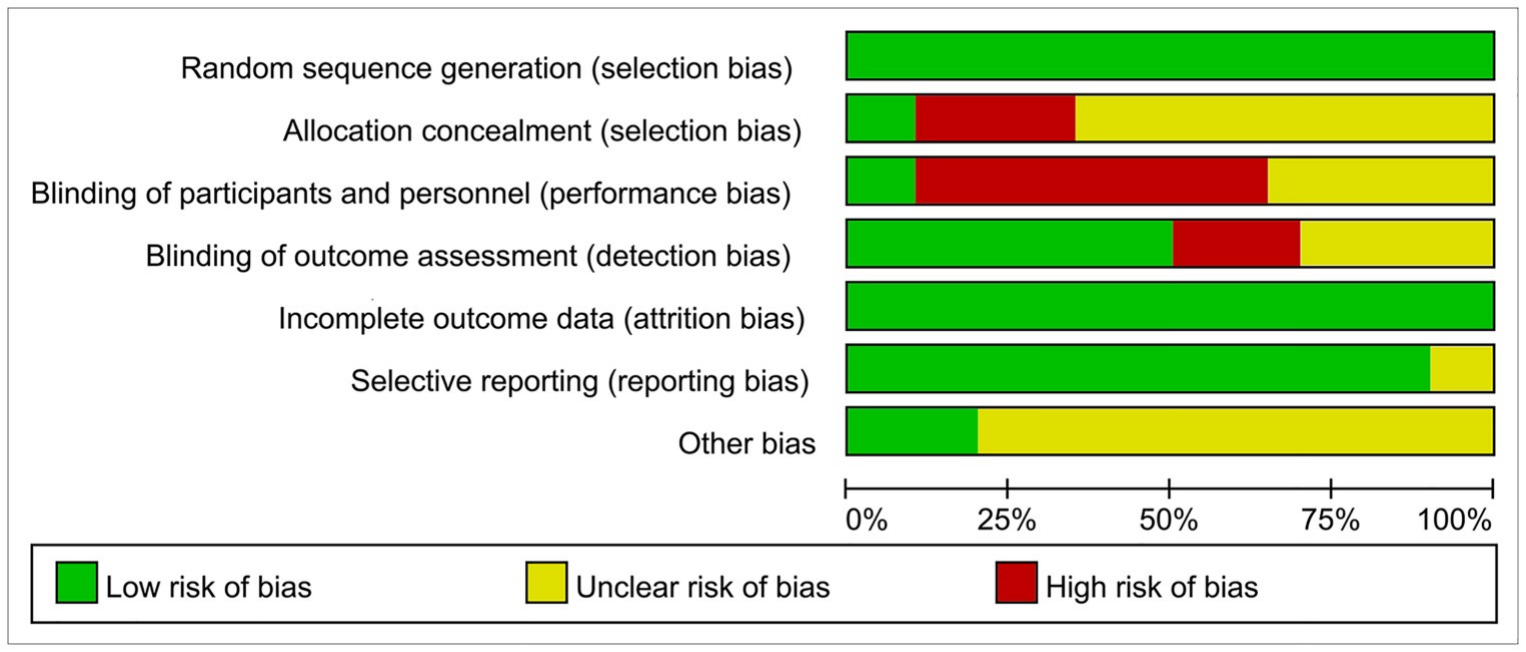
Risk of bias graph: A review of the authors’ judgments about each risk of bias item presented as percentages across all included studies.

**Fig 3:**
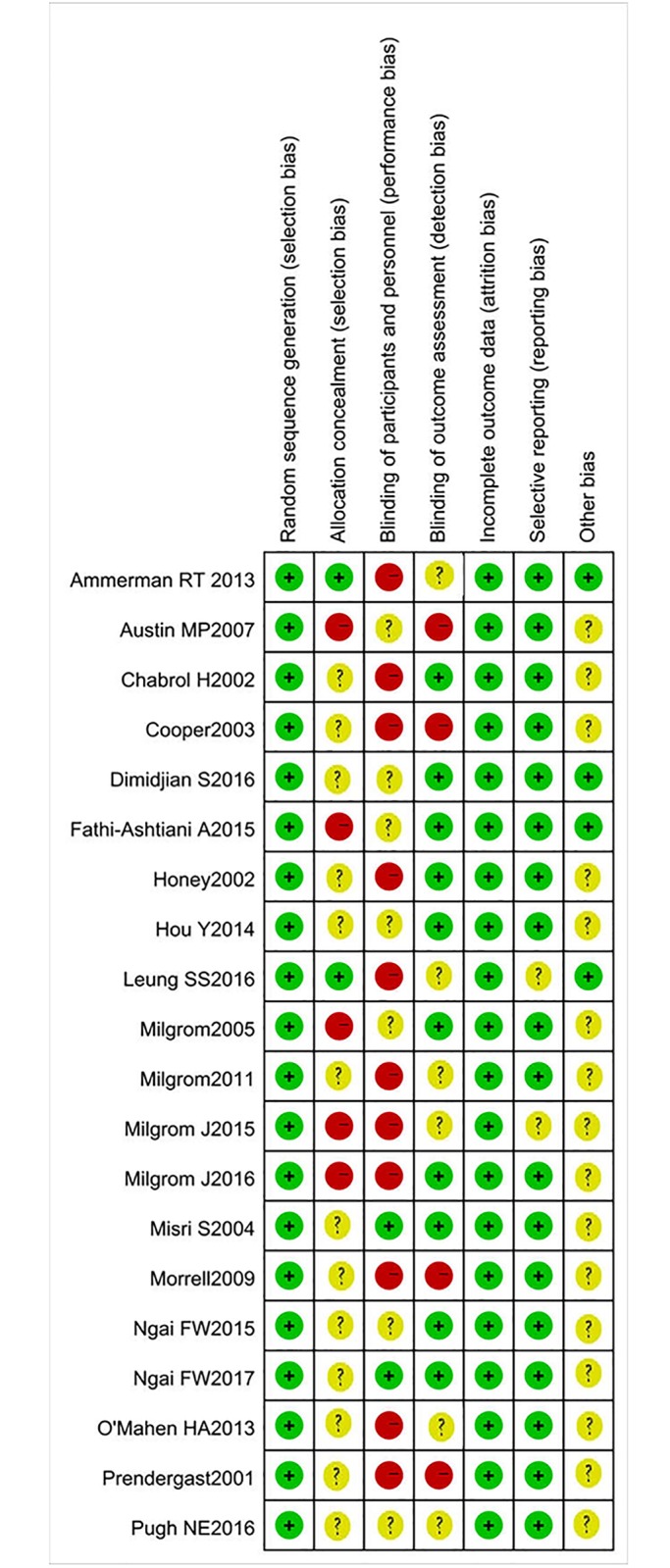
Summary of risk of bias: A review of the authors’ judgments about each risk of bias item in each included study.

Although randomization was reported in all included RCTs, few studies [27, 33, 39, 42] reported specific method of randomization. It was impossible to set blind for patients due to the theory of the intervention, however, some studies [27, 39] reported that blind was applied in the study design. We regarded high-performance bias in these trials. Due to the critical requirements or missing data of the allocation concealment, most of the included studies were considered as high-risk or unclear risk of allocation concealment. Overall, according to the risk assessment criteria of the Cochrane Handbook, more than 60% of the studies were considered as low-risk of bias, approximately 30% as unclear risk, and less than 10% as high-risk of bias (Figs 2 and 3).

### Effectiveness of interventions

#### PND symptoms

**Fig 4:**
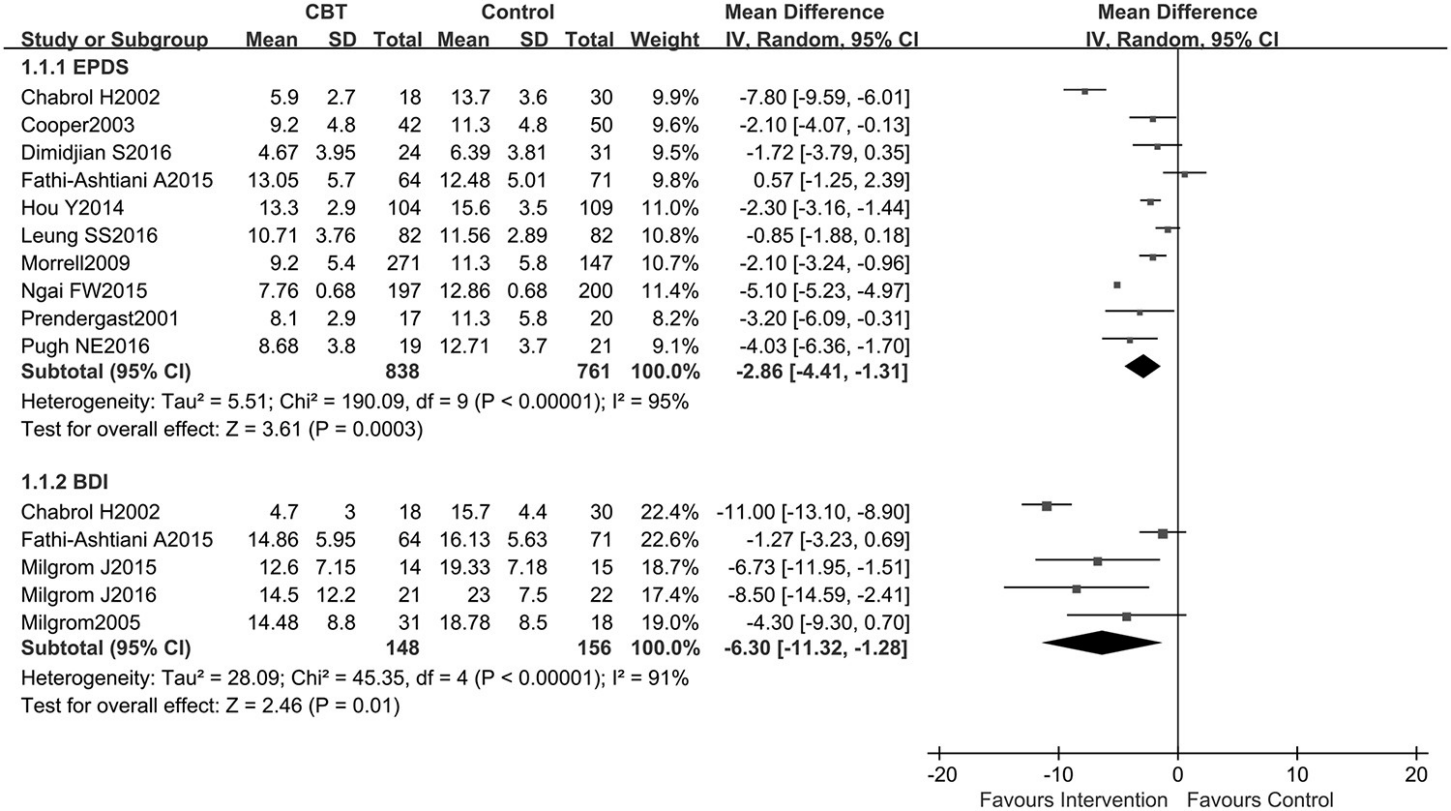
Combined results of EPDS and BDI scores comparing CBT vs. control conditions immediately post-intervention: A random effects analysis.

**Fig 5:**
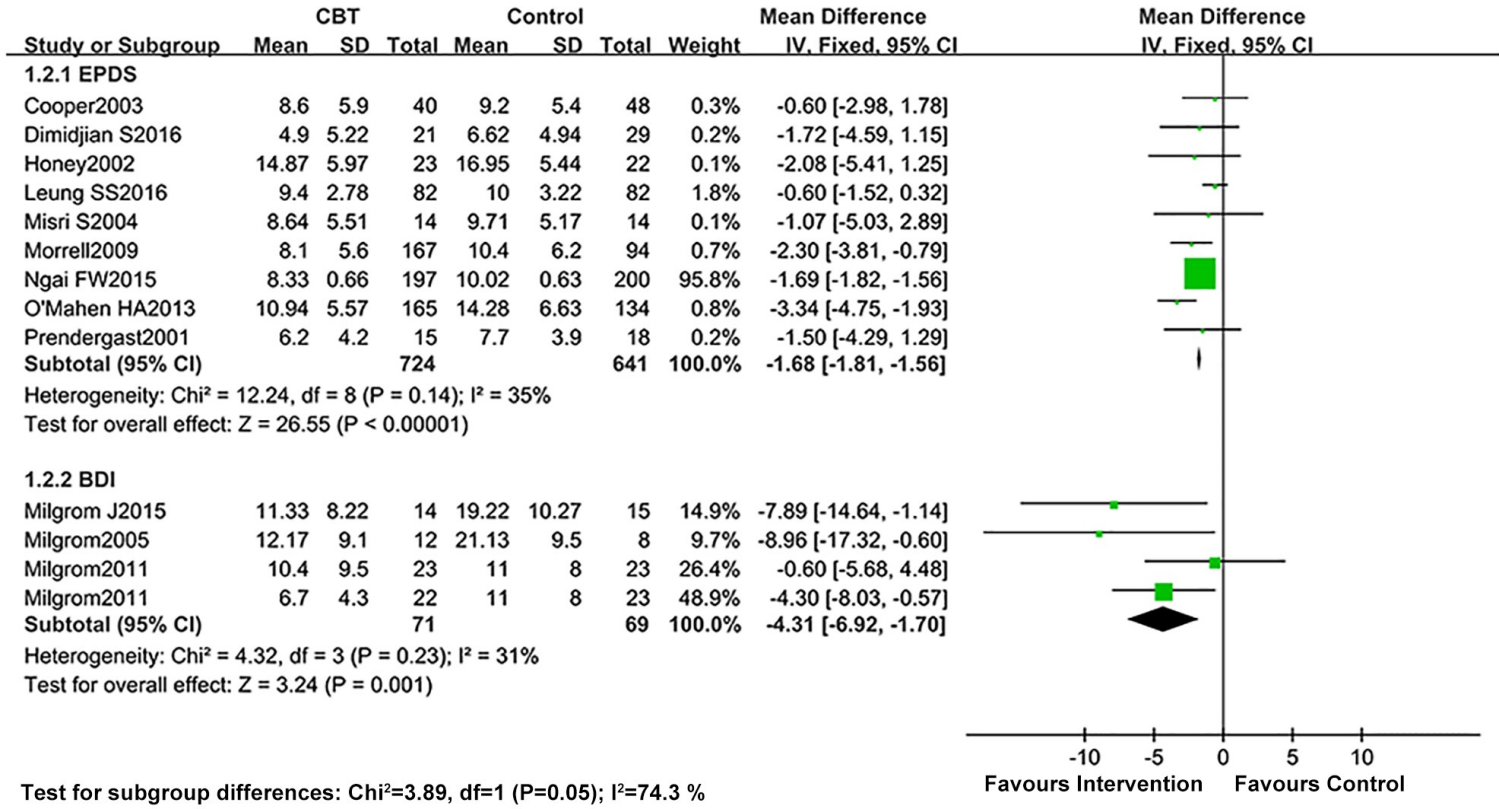
Combined results of EPDS and BDI scores comparing CBT vs. control conditions at long-term post-intervention: A random effects analysis.

A total of 13 studies reported the data pertaining the improvement of depressive symptoms after interventions as assessed by either EPDS or BDI. Heterogeneity detection results did not suggest a significant heterogeneity (I^2^<40%) for long-term evaluation, and the fixed effects model was introduced for this part of the meta-analysis. After CBT, the scores of EPDS in the intervention group were significantly lower than those in the control group, and the differences in both short- and long-term effects were statistically significant (short-term: SMD = -2.86, 95% CI: -4.41–-1.31; *P*<0.05, and long-term: SMD = -1.68, 95% CI: -1.81–-1.56; *P*<0.05). The results are presented in Figs 4 and 5. While assessing the effect of CBT on short-term of BDI, the random effects model was applied owing to significant heterogeneity (I^2^>50%). The results of meta-analysis showed that the scores of BDI in the CBT group reduced more than those of the control group, both in short-term (mean difference = -6.30, 95% CI: -11.32–-1.28; *P*<0.05) and long-term (mean difference = -4.31, 95% CI: -6.92–-1.70; *P*<0.05). The studies on sensitivity analysis by Ngai et al. [31] and Morrell et al. [36] were the main sources of heterogeneity (S1 Fig). The study of Fathi-Ashtiani et al. [30] was considered as the primary source of heterogeneity for pooled analysis of short-term BDI (S2 Fig). After removing this study, the I^2^ decreased from 91 to 58%.

**Fig 6:**
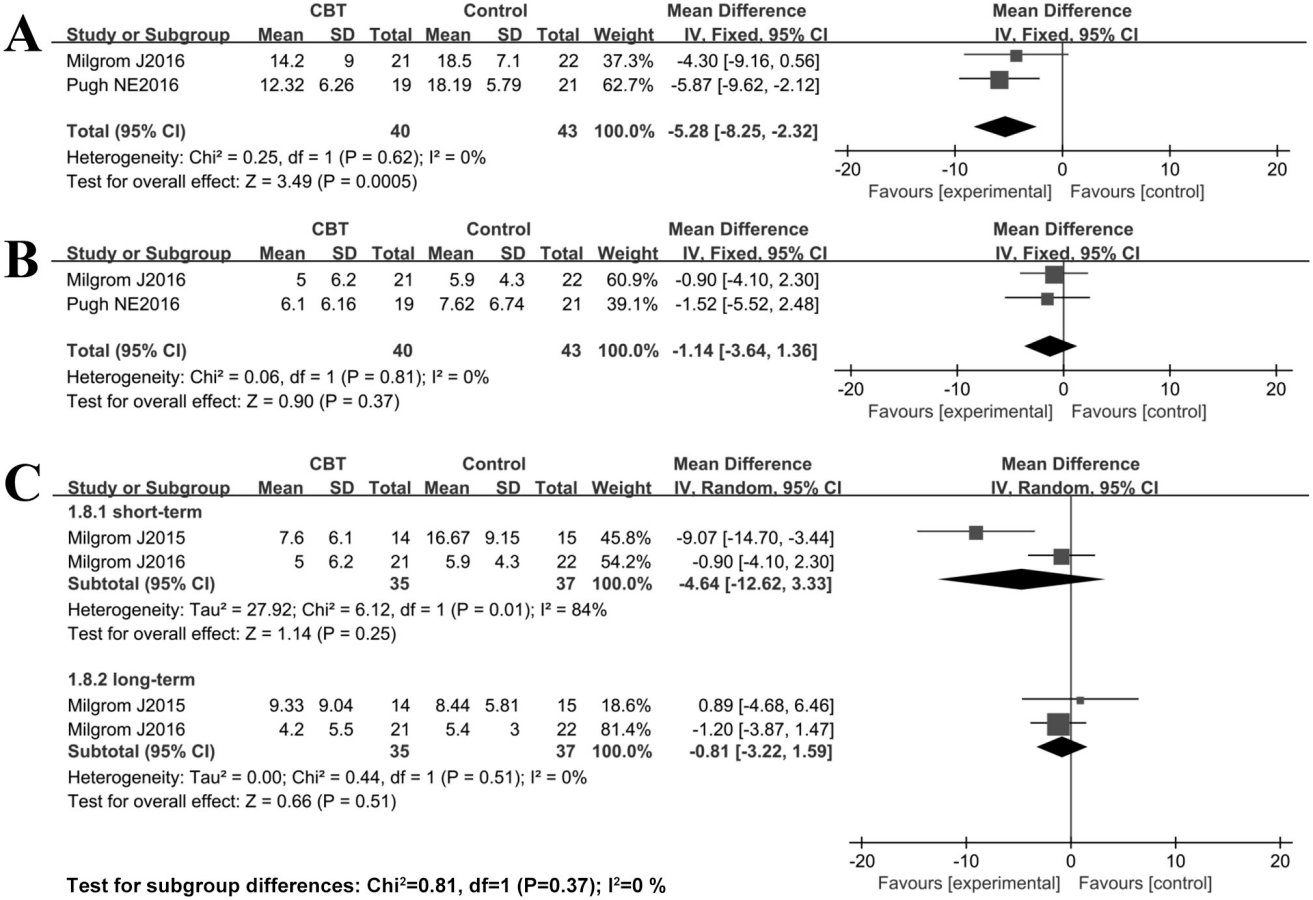
Forest plot of DASS and BAI scores comparing CBT vs. control conditions post-intervention: A random effects analysis. A, DASS-stress; B, DASS-anxiety; C, BAI -anxiety.

Other indicators of depression and/or anxiety were also evaluated, although only three RCTs reported data regarding BAI and DASS. No significant heterogeneity was observed as indicated by the I^2^%, and thus, the fixed effects model was used for DASS analysis, but not for BAI (I^2^>50%). The results of meta-analysis showed that the scores of BAI in the intervention group were lower than those in the control group; however, the differences of short-term (SMD = -4.64, 95% CI: -12.62–3.33; *P* = 0.25) and long-term (SMD = -0.81, 95% CI: -3.22–1.59; *P* = 0.51) were not statistically significant. On the other hand, the combined results showed that the scores of DASS in the intervention group were lower than those in the control group, and the difference of DASS-stress (SMD = -5.28, 95% CI: -8.25–-2.32; *P*<0.01) was statistically significant, except for DASS-anxiety (SMD = -1.14, 95% CI: -3.64–1.36; *P* = 0.37). The detailed results are illustrated in Fig 6.

### Subgroup analysis based on the types of intervention

As several types of therapies were used among these included studies, we performed a subgroup analysis to determine the most optimal intervention that exerted the best effect on relieving symptoms of depression with respect to EPDS. The results showed that in-home-based CBT was significantly superior in improving depression in short-term (SMD = -0.97, 95% CI: -1.81–-0.13; *P*<0.01) and long-term (SMD = -0.31, 95% CI: -0.57–-0.05; *P*<0.01) as compared to the control group. Internet-based and telephone-based therapies were also efficient; however, the results were not convincing owing to the limited number of included studies (S3 and S4 Figs).

### Improvement of postnatal depression after treatments

**Fig 7:**
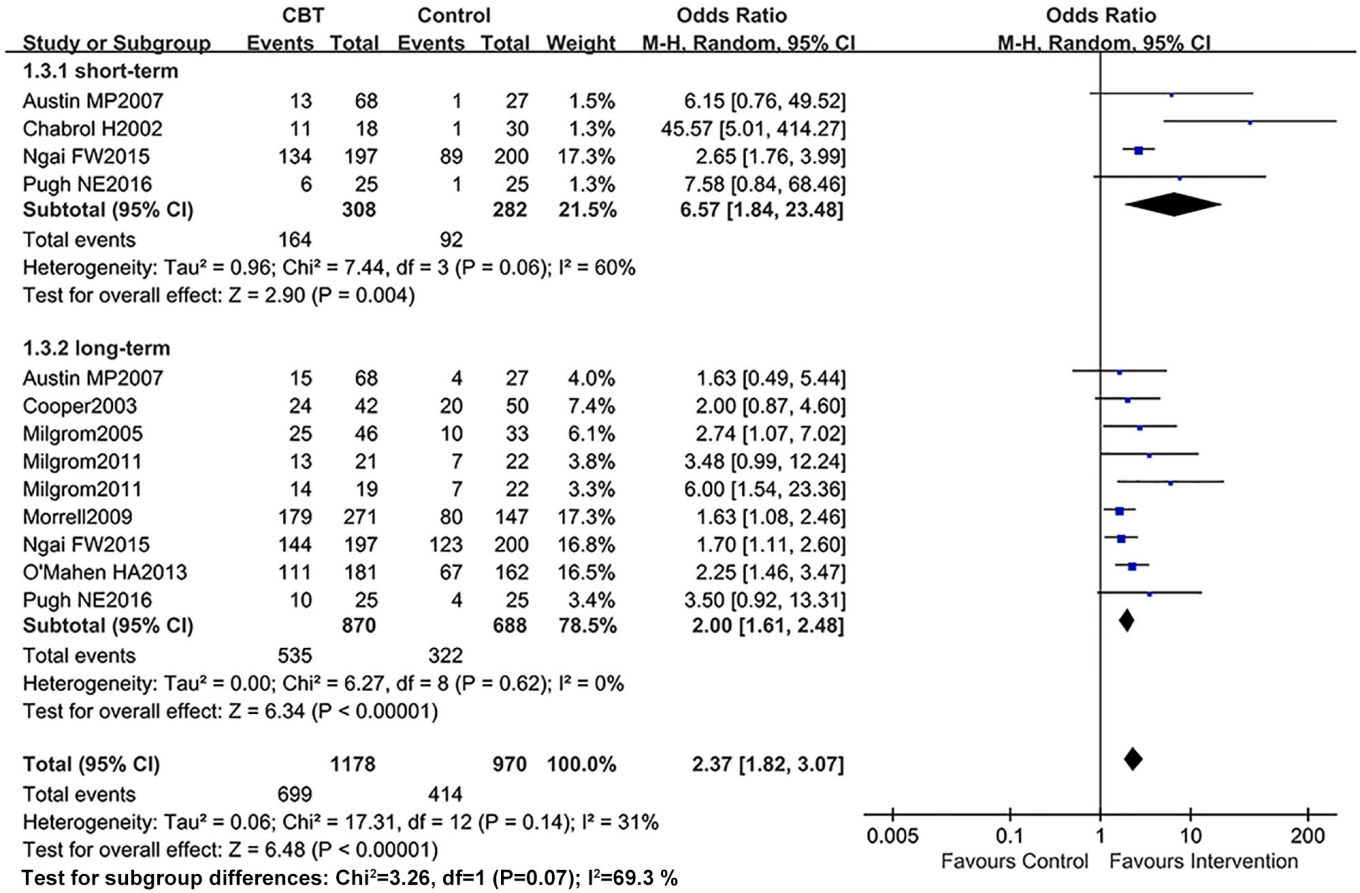
Meta-analysis of the OR in women who improved and failed to meet the diagnostic criteria (variously defined) comparing CBT with control post-intervention: A random effects analysis.

Ten RCTs reported women under the diagnostic criteria after interventions. As there was significant heterogeneity (I^2^>50%), the random effects model was introduced during this part of the meta-analysis. As shown in Fig 7, after CBT, the number of women cured of PND in the intervention group was significantly higher than those in the control group, the differences of short-term (OR = 6.57, 95% CI: 1.84–23.48; *P*<0.05) and long-term (OR = 2.00, 95% CI: 1.61–2.48; *P*<0.05) were both statistically significant.

### Publication bias

**Fig 8:**
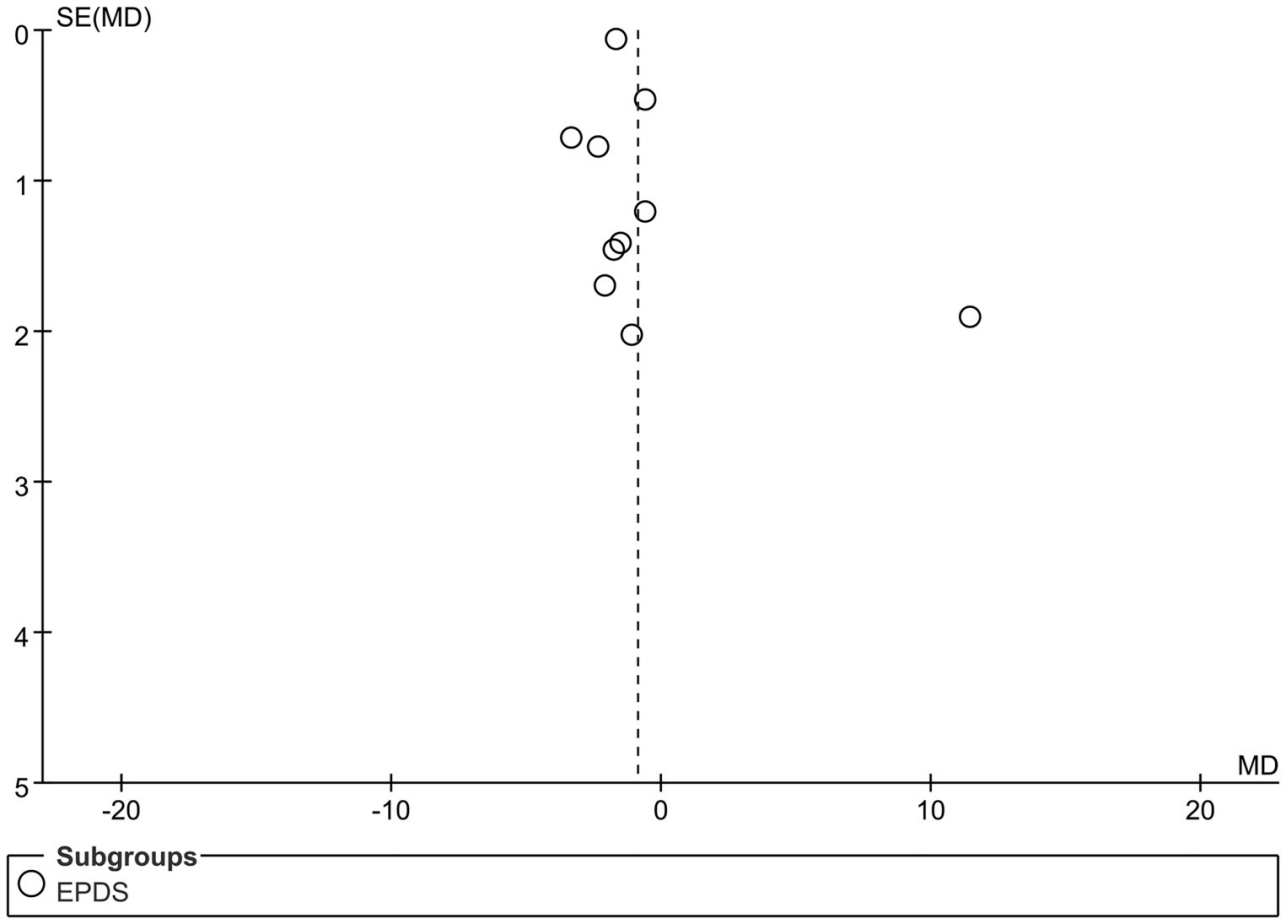
Funnel plots for evaluating the publication bias.

The publication bias of the included studies in EPDS was evaluated using the inverted funnel plots and quantified using the Egger’s test and Begg’s test. Fig 8 demonstrated a satisfactorily symmetrical inverted funnel plot (Egger’s test: *P* = 0.802>0.05; Begg’s test: *P* = 0.858>0.05), suggesting no significant bias of publication.

## Discussion

Meta-analysis is an important method for evidence-based medicine [26]. It refers to the systematic, objective, and comprehensive quantitative analysis of results from multiple independent studies with identical objectives by appropriate statistical methods. In this study, 20 RCTs were included in the meta-analysis. The results showed that CBT was clinically more effective than conventional therapies, with a reduced number of PND patients and improved symptoms of the disease. Notably, multiple therapies including CBT and anti-depressive medication were used in the included studies, making the effects of therapies difficult to separate from each other.

Recently, several meta-analyses [2, 9, 19, 22] assessed the reliable and effective therapy for PND, and the results were not consistent. In 2013, Scope et al. [44] performed a meta-analysis to evaluate whether CBT could serve as an evidence-based treatment for women with PND as compared to other used packages of care. They identified 7 eligible studies for the final meta-analysis, which showed that the CBT group could be effective in reducing depression when compared with the usual care, routine primary care, or waiting list groups. However, all the included studies were not RCTs, and the varied definitions of the CBT component of the studies suggested that the results should be interpreted cautiously. Morrell et al. [9] systematically evaluated the clinical effectiveness and cost-effectiveness of postnatal intervention in women to prevent PND. The meta-analysis designated CBT as the most beneficial intervention. The study concluded that cognitive behavioral therapy was cost-effective with respect to traditional care, although much of the evidence was inconclusive. The present study focused primarily on the cost-efficiency of CBT; the clinical efficiency of the methods has not been assessed systematically. Furthermore, Lau et al. [19] performed another meta-analysis to evaluate the efficacy of the therapist-supported internet-based CBT on postpartum women. After searching, a total of 8 RCTs involving 1523 participants across six nations were included. They found that this therapy could significantly improve stress, anxiety, and depressive symptoms in postpartum depressive women as compared to those of the control group. Though the results were positive, the therapist-supported internet-based cognitive behavior therapy was only one specific type of the cognitive behavior therapy. Although these studies determined that CBT was useful in improving the depressive symptoms among women with postnatal depression. However, these meta-analyses did not systematically assess the efficiencies of different types of CBT in treating postpartum women as compared to control groups.

Our meta-analysis found that the CBT, overall or in different forms, had significant benefits in treating women with PND. The overall results of our meta-analysis showed that the CBT exerted good short-term and long-term effects on PND. After the administration of intervention, the symptoms of PND patients could be significantly improved immediately after treatment within few weeks and maintained for >3 months. Non-guided counseling and usual therapy had only a good short-term effect on PND. At the end of the intervention, these methods could significantly improve the symptoms; however, the long-term efficacy was poor. In-home-based, internet-based, and telephone-based exhibited a good short-term effect on PND, and the long-term efficacy remains to be further elucidated. The sub-group analyses showed that the internet-based therapy, telephone-based therapy, in-home therapy, mindfulness therapy, and other forms of CBT were efficacious in relieving symptoms and reducing the number of PND patients. These findings indicated that CBT could exert its therapeutic role in various forms of delivery, enabling the participants to select appropriate therapeutic pathway, thereby increasing the treatment compliance.

This meta-analysis aimed to systematically evaluate the efficiencies of CBT in treating new mothers with PND based on the latest clinical evidence. In the current study, the sub-group analysis was introduced to determine the efficacy of different interventions and forms of therapy delivery. The outcomes of symptoms improvement and short- and long-term effects were also analyzed in our meta-analysis, further strengthening the findings of this meta-analysis. However, there were several limitations within our meta-analysis. First, the number and samples of included RCTs were relatively small, especially in the sub-group analysis. Second, heterogeneity was observed among included studies with respects to study design, treatments, indicators of outcomes, and level of detail of the follow-up. The relapse rate of PND differed between studies. They reported different duration and improvements of depression symptoms. The period of follow-up was different among the included studies, which might over- or under-estimate the actual effectiveness of CBT. Third, the language of eligible studies was primarily limited to English, which might lead to the risk of selection bias. Nevertheless, the results of this meta-analysis showed that CBT was effective in treating PND, both in improving symptoms and reducing the number of patients suffering from the disorder.

## Conclusion

In conclusion, the cognitive behavioral therapy is sufficient to relieve the psychological symptoms of postnatal depression and effectively improve the quality of life in new mothers, resulting in a reduced prevalence of postnatal depression. However, our findings are limited due to the uneven quality, the differences in the diagnostic criteria, evaluation methods, and follow-up of the included studies. Thus, rigorous high-quality randomized controlled clinical trials are essential to substantiate the present conclusion.

## Supporting information

S1 Fig Sensitivity analysis of short-term EPDS. (TIF)

S2 Fig Sensitivity analysis of short-term BDI. (TIF)

S3 Fig Subgroup analysis of short-term of EPDS. (TIF)

S4 Fig Subgroup analysis of long-term of EPDS. (TIF)

S1 Table PRISMA checklist. (DOC)

S1 File Data. Raw data.
(XLSX)

## References

[1] Verbeek T, Geelhoed-Jenner BNWJ, Nijhuis ER, Peer CD, Berger MY. Psychopathology during and after pregnancy. Nederlands Tijdschrift voor Geneeskunde. 2015; 159(42):26420148

[2] Stephens S, Ford E, Paudyal P, Smith H. Effectiveness of Psychological Interventions for Postnatal Depression in Primary Care: A Meta-Analysis. Ann Fam Med. 2016; 14(5): 463–472. 10.1370/afm.1967 27621164 PMC5394369

[3] Bais B, Kamperman AM, van der Zwaag MD, Dieleman GC, Harmsen van der Vliet-Torij HW, Bijma HH, et al Bright light therapy in pregnant women with major depressive disorder: study protocol for a randomized, double-blind, controlled clinical trial. BMC Psychiatry. 2016; 16(1): 381 10.1186/s12888-016-1092-2 27821114 PMC5100252

[4] Drury SS, Scaramella L, Zeanah CH. The Neurobiological Impact of Postpartum Maternal Depression: Prevention and Intervention Approaches. Child and Adolescent Psychiatric Clinics of North America. 2016; 25(2): 179–200. 10.1016/j.chc.2015.11.001 26980123 PMC4794751

[5] de Camps Meschino D, Philipp D, Israel A, Vigod S. Maternal-infant mental health: postpartum group intervention. Arch Womens Ment Health. 2016; 19(2): 243–251. 10.1007/s00737-015-0551-y 26239582

[6] Marino C, Riva V, Mornati G, Nobile M, Vanzin L, Dionne G, et al Paternal postnatal depression affects 6-month-old infants development. Early Intervention in Psychiatry. 2016; 10 102.

[7] Salari AA, Fatehi-Gharehlar L, Motayagheni N, Homberg JR. Fluoxetine normalizes the effects of prenatal maternal stress on depression- and anxiety-like behaviors in mouse dams and male offspring. Behavioural Brain Research. 2016; 311 354–367. 10.1016/j.bbr.2016.05.062 27263073

[8] Tomfohr-Madsen LM, Campbell TS, Giesbrecht GF, Letourneau NL, Carlson LE, Madsen JW, et al Mindfulness-based cognitive therapy for psychological distress in pregnancy: study protocol for a randomized controlled trial. Trials. 2016; 17(1): 498 10.1186/s13063-016-1601-0 27737714 PMC5064936

[9] Morrell CJ, Sutcliffe P, Booth A, Stevens J, Scope A, Stevenson M, et al A systematic review, evidence synthesis and meta-analysis of quantitative and qualitative studies evaluating the clinical effectiveness, the cost-effectiveness, safety and acceptability of interventions to prevent postnatal depression. Health Technol Assess. 2016; 20(37): 1–414. 10.3310/hta20370 27184772 PMC4885009

[10] Dixon S, Dantas JA. Best practice for community based management of postnatal depression in developing countries: a systematic review. Health Care Women Int. 2016; 0. 10.1080/07399332.2016.1255213 27918855

[11] Usuda K, Nishi D, Makino M, Tachimori H, Matsuoka Y, Sano Y, et al Prevalence and related factors of common mental disorders during pregnancy in Japan: a cross-sectional study. Biopsychosoc Med. 2016; 10 17. 27213012 10.1186/s13030-016-0069-1 PMC4874014

[12] Wald MF, Muzyk AJ, Clark D. Bipolar Depression: Pregnancy, Postpartum, and Lactation. Psychiatric Clinics of North America. 2016; 39(1): 57–74. 10.1016/j.psc.2015.10.002 26876318

[13] MacQueen GM, Frey BN, Ismail Z, Jaworska N, Steiner M, Lieshout RJV, et al Canadian Network for Mood and Anxiety Treatments (CANMAT) 2016 clinical guidelines for the management of adults with major depressive disorder: Section 6. Special populations: Youth, women, and the elderly. Canadian Journal of Psychiatry. 2016; 61(9): 588–603. 10.1177/0706743716659276 27486149 PMC4994788

[14] Hashmi AM, Bhatia SK, Bhatia SK, Khawaja IS. Insomnia during pregnancy: Diagnosis and rational interventions. Pakistan Journal of Medical Sciences. 2016; 32(4): 1030–1037. doi: 10.12669/pjms.324.10421 27648062 10.12669/pjms.324.10421 PMC5017073

[15] Milgrom J, Gemmill AW, Ericksen J, Burrows G, Buist A, Reece J. Treatment of postnatal depression with cognitive behavioural therapy, sertraline and combination therapy: a randomised controlled trial. Aust N Z J Psychiatry. 2015; 49(3): 236–245. 10.1177/0004867414565474 25586754

[16] Guille C, Newman R, Fryml LD, Lifton CK, Epperson CN. Management of postpartum depression. Journal of Midwifery and Women’s Health. 2013; 58(6): 632–642. 10.1111/jmwh.12104 PMC4101986 24131708

[17] Stuart S, Koleva H. Psychological treatments for perinatal depression. Best Practice and Research: Clinical Obstetrics and Gynaecology. 2014; 28(1): 61–70. 10.1016/j.bpobgyn.2013.09.004 24269903

[18] Bozkurt O, Eras Z, Sari FN, Dizdar EA, Uras N, Canpolat FE, et al Does maternal psychological distress affect neurodevelopmental outcomes of preterm infants at a gestational age of ≤ 32 weeks. Early Human Development. 2017; 104 27–31. 10.1016/j.earlhumdev.2016.11.006 27978476

[19] Lau Y, Htun TP, Wong SN, Tam WSW, Klainin-Yobas P. Therapist-Supported Internet-Based Cognitive Behavior Therapy for Stress, Anxiety, and Depressive Symptoms Among Postpartum Women: A Systematic Review and Meta-Analysis. J Med Internet Res. 2017; 19(4): e138 10.2196/jmir.6712 28455276 PMC5429436

[20] Milgrom J, Danaher BG. Internet Cognitive Behavioral Therapy for Women With Postnatal Depression: A Randomized Controlled Trial of MumMoodBooster. 2016; 18(3): e54. 10.2196/jmir.4993 PMC4802107 26952645

[21] Ericksen J, Pugh NE, Hadjistavropoulos HD, Dirkse D. A Randomised Controlled Trial of Therapist-Assisted, Internet-Delivered Cognitive Behavior Therapy for Women with Maternal Depression. J Med Internet Res. 2016; 11(3): e0149186. 10.1371/journal.pone.0149186 PMC4773121 26930488

[22] Lever Taylor B, Cavanagh K, Strauss C. The Effectiveness of Mindfulness-Based Interventions in the Perinatal Period: A Systematic Review and Meta-Analysis. PLoS One. 2016; 11(5): e0155720 10.1371/journal.pone.0155720 27182732 PMC4868288

[23] Yazdanimehr R, Omidi A, Sadat Z, Akbari H. The Effect of Mindfulness-integrated Cognitive Behavior Therapy on Depression and Anxiety among Pregnant Women: a Randomized Clinical Trial. J Caring Sci. 2016; 5(3): 195–204. doi: 10.15171/jcs.2016.021 27752485 10.15171/jcs.2016.021 PMC5045953

[24] Ngai FW, Wong PW, Chung KF, Leung KY. The effect of telephone-based cognitive-behavioural therapy on parenting stress: A randomised controlled trial. J Psychosom Res. 2016; 86 34–38. 10.1016/j.jpsychores.2016.03.016 27302544

[25] Kammerer M, Castro RTA, Glover V, Ramchandani P, Bennett P. A randomized controlled trial of internet based cognitive behavioural therapy (CBT) versus treatment as usual for pregnant women with high levels of depression at queen charlotte’s hospital. Archives of Women’s Mental Health. 2013; 16 S18.

[26] Marsh N, Webster J, Mihala G, Rickard CM. Devices and dressings to secure peripheral venous catheters: A Cochrane systematic review and meta-analysis. Int J Nurs Stud. 2017; 67 12–19. 10.1016/j.ijnurstu.2016.11.007 27889585

[27] Ngai FW, Wong PW, Chung KF, Leung KY. The effect of a telephone-based cognitive behavioral therapy on quality of life: a randomized controlled trial. Arch Womens Ment Health. 2017; 10.1007/s00737-017-0722-0 28361441

[28] Leung SS, Lee AM, Wong DF, Wong CM, Leung KY, Chiang VC, et al A brief group intervention using a cognitive-behavioural approach to reduce postnatal depressive symptoms: a randomised controlled trial. Hong Kong Med J. 2016; 22 Suppl 2 S4–8. 26908335

[29] Dimidjian S, Goodman SH, Felder JN, Gallop R, Brown AP, Beck A. Staying well during pregnancy and the postpartum: A pilot randomized trial of mindfulness-based cognitive therapy for the prevention of depressive relapse/recurrence. J Consult Clin Psychol. 2016; 84(2): 134–145. 10.1037/ccp0000068 26654212 PMC5718345

[30] Fathi-Ashtiani A, Ahmadi A, Ghobari-Bonab B, Azizi MP, Saheb-Alzamani SM. Randomized Trial of Psychological Interventions to Preventing Postpartum Depression among Iranian First-time Mothers. Int J Prev Med. 2015; 6 109 10.4103/2008-7802.169078 26682030 PMC4671165

[31] Ngai FW, Wong PW, Leung KY, Chau PH, Chung KF. The Effect of Telephone-Based Cognitive-Behavioral Therapy on Postnatal Depression: A Randomized Controlled Trial. Psychother Psychosom. 2015; 84(5): 294–303. 10.1159/000430449 26278623

[32] Hou Y, Hu P, Zhang Y, Lu Q, Wang D, Yin L, et al Cognitive behavioral therapy in combination with systemic family therapy improves mild to moderate postpartum depression. Rev Bras Psiquiatr. 2014; 36(1): 47–52. 10.1590/1516-4446-2013-1170 24604461

[33] O’Mahen HA, Woodford J, McGinley J, Warren FC, Richards DA, Lynch TR, et al Internet-based behavioral activation—treatment for postnatal depression (Netmums): a randomized controlled trial. J Affect Disord. 2013; 150(3): 814–822. 10.1016/j.jad.2013.03.005 23602514

[34] Ammerman RT, Putnam FW, Altaye M, Teeters AR, Stevens J, Van Ginkel JB. Treatment of depressed mothers in home visiting: impact on psychological distress and social functioning. Child Abuse Negl. 2013; 37(8): 544–554. 10.1016/j.chiabu.2013.03.003 23623623 PMC3742640

[35] Milgrom J, Holt CJ, Gemmill AW, Ericksen J, Leigh B, Buist A, et al Treating postnatal depressive symptoms in primary care: a randomised controlled trial of GP management, with and without adjunctive counselling. BMC Psychiatry. 2011; 11 95 10.1186/1471-244X-11-95 21615968 PMC3121669

[36] Morrell CJ, Slade P, Warner R, Paley G, Dixon S, Walters SJ, et al Clinical effectiveness of health visitor training in psychologically informed approaches for depression in postnatal women: pragmatic cluster randomised trial in primary care. Bmj. 2009; 338 a3045 10.1136/bmj.a3045 19147636 PMC2628298

[37] Austin MP, Frilingos M, Lumley J, Hadzi-Pavlovic D, Roncolato W, Acland S, et al Brief antenatal cognitive behaviour therapy group intervention for the prevention of postnatal depression and anxiety: a randomised controlled trial. J Affect Disord. 2008; 105(1–3): 35–44. 10.1016/j.jad.2007.04.001 17490753

[38] Milgrom J, Negri LM, Gemmill AW, McNeil M, Martin PR. A randomized controlled trial of psychological interventions for postnatal depression. Br J Clin Psychol. 2005; 44(Pt 4): 529–542. 10.1348/014466505X34200 16368032

[39] Misri S, Reebye P, Corral M, Milis L. The use of paroxetine and cognitive-behavioral therapy in postpartum depression and anxiety: a randomized controlled trial. J Clin Psychiatry. 2004; 65(9): 1236–1241. 15367052 10.4088/jcp.v65n0913

[40] Cooper PJ, Murray L, Wilson A, Romaniuk H. Controlled trial of the short- and long-term effect of psychological treatment of post-partum depression. I. Impact on maternal mood. Br J Psychiatry. 2003; 182 412–419. 12724244

[41] Honey KL, Bennett P, Morgan M. A brief psycho-educational group intervention for postnatal depression. Br J Clin Psychol. 2002; 41(Pt 4): 405–409. 12437794 10.1348/014466502760387515

[42] Chabrol H, Teissedre F, Saint-Jean M, Teisseyre N, Roge B, Mullet E. Prevention and treatment of post-partum depression: a controlled randomized study on women at risk. Psychol Med. 2002; 32(6): 1039–1047. 12214785 10.1017/s0033291702006062

[43] Prendergast J, Austin MP. Early childhood nurse-delivered cognitive behavioural counselling for post-natal depression. Australasian Psychiatry. 2001; 9(3): 255–259.

[44] Scope A, Leaviss J, Kaltenthaler E, Parry G, Sutcliffe P, Bradburn M, et al Is group cognitive behaviour therapy for postnatal depression evidence-based practice? A systematic review. BMC Psychiatry. 2013; 13 321 10.1186/1471-244X-13-321 24283266 PMC4219505

