# Supplementary figures and images for: Is cognitive behavioral therapy a better choice for women with postnatal depression? A systematic review and meta-analysis
Source: PLoS One. 2018 Oct 15;13(10):e0205243. doi: 10.1371/journal.pone.0205243 (PMC6188757; doi:10.1371/journal.pone.0205243)

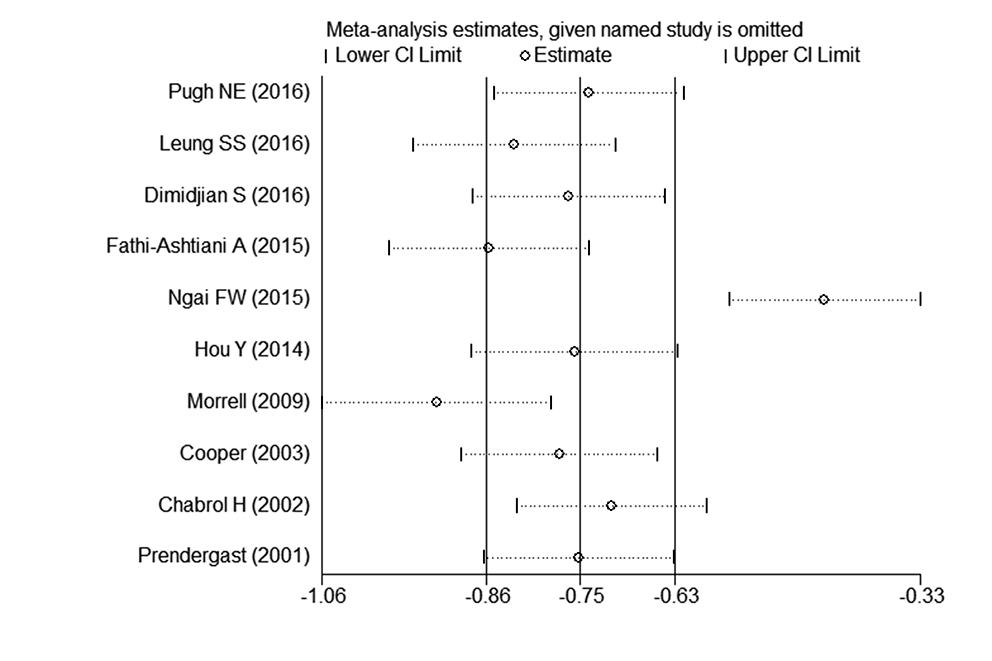

Supplement: S1 Fig — (TIF) [file pone.0205243.s001.tif]

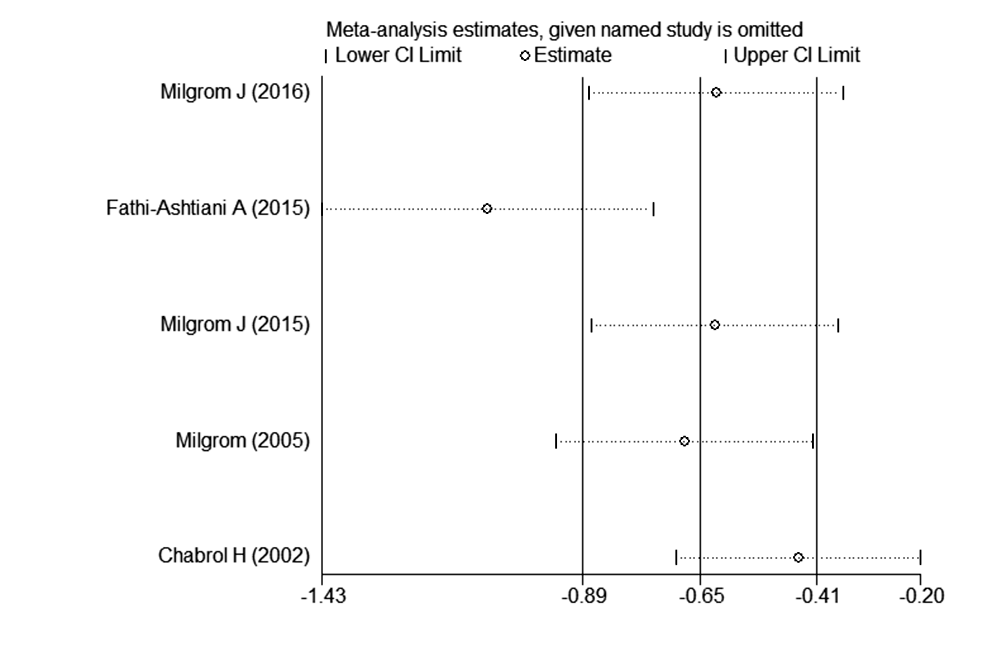

Supplement: S2 Fig — (TIF) [file pone.0205243.s002.tif]

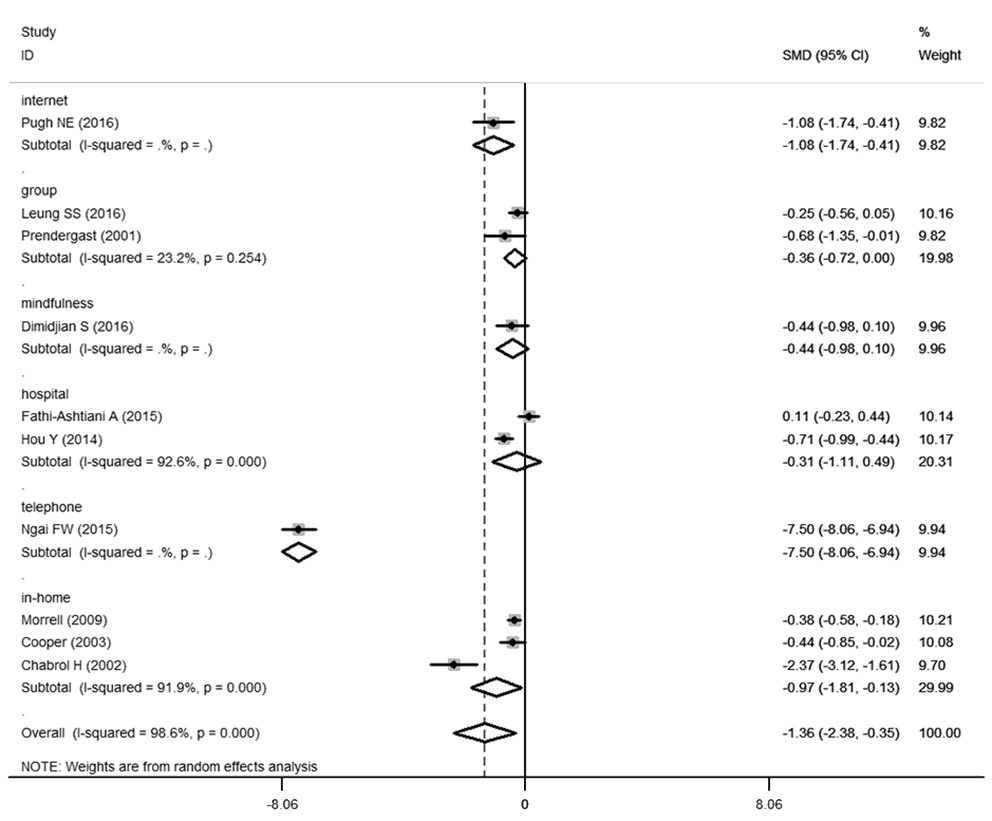

Supplement: S3 Fig — (TIF) [file pone.0205243.s003.tif]

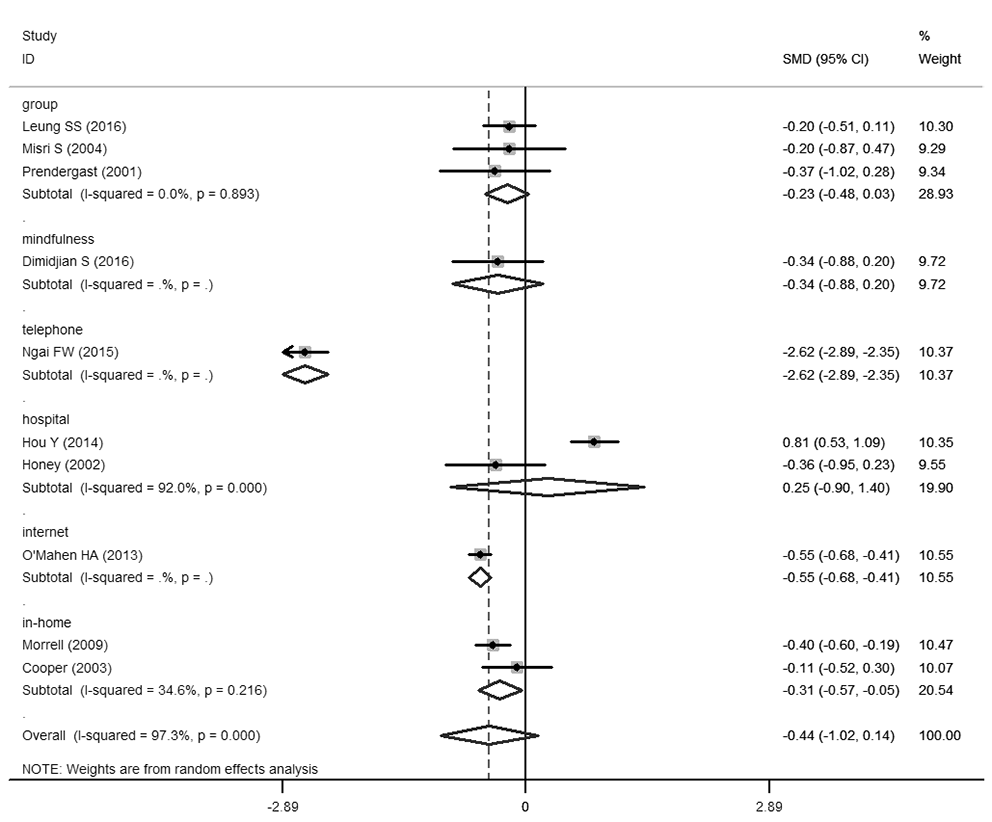

Supplement: S4 Fig — (TIF) [file pone.0205243.s004.tif]
